# Assessment of Maternal Glycemia and Newborn Size Among Pregnant Women Who Use Wood Stoves in Northern New England

**DOI:** 10.1001/jamanetworkopen.2020.6046

**Published:** 2020-05-28

**Authors:** Abby F. Fleisch, Shravanthi M. Seshasayee, Eric Garshick, Jonathan W. Chipman, Petros Koutrakis, Emily R. Baker, Margaret R. Karagas

**Affiliations:** 1 Division of Diabetes & Endocrinology, Pediatric Specialty Care, Maine Medical Partners, Portland; 2 Center for Outcomes Research and Evaluation, Maine Medical Center Research Institute, Portland; 3 Pulmonary, Allergy, Sleep, and Critical Care Medicine Section, VA Boston Healthcare System, Jamaica Plain, Massachusetts; 4 Channing Division of Network Medicine, Department of Medicine, Brigham and Women’s Hospital, Harvard Medical School, Boston, Massachusetts; 5 Department of Geography, Dartmouth College, Hanover, New Hampshire; 6 Department of Environmental Health, Harvard T.H. Chan School of Public Health, Boston, Massachusetts; 7 Department of Epidemiology, Geisel School of Medicine at Dartmouth, Hanover, New Hampshire; 8 Children’s Environmental Health and Disease Prevention Research Center at Dartmouth, Hanover, New Hampshire

## Abstract

This cohort study uses data from the New Hampshire Birth Cohort Study to assess maternal glycemia and newborn size among pregnant women who use wood-burning stoves in their New England homes.

## Introduction

Wood stove use has increased in the United States, with over 12 million households using wood as a primary or supplemental heat source.^1^ Although wood smoke from cooking stoves has been studied in developing countries, little is known about the health of individuals who use domestic wood stoves, particularly during pregnancy. Here, we evaluate the association of wood stove use during pregnancy with maternal glycemia and infant birth size in a large cohort in northern New England.

## Methods

Women were recruited into the New Hampshire Birth Cohort Study at approximately 24-28 weeks’ gestation beginning in January 2009; the study is currently ongoing. At the time of this analysis, data were available on 1977 participants, and we studied 1223 (61.9%) who had exposure, outcome, and covariate information and did not use a fireplace or have preexisting diabetes. Women included in our cohort study were similar to the overall cohort with respect to sociodemographic characteristics, glucose tolerance, and birth size. The Committee for the Protection of Human Subjects at Dartmouth College approved the New Hampshire Birth Cohort Study, and all the participants provided written informed consent. The institutional review board of the Maine Medical Center determined the present study to be exempt from review because it is a secondary analysis of previously collected, deidentified data.

Women reported sociodemographic information and household wood stove use on questionnaires at the time of enrollment and at 2 weeks postpartum. We extracted neighborhood wood stove use and home roadway proximity from US Census data. We classified women as having normal glucose tolerance, impaired glucose tolerance, or gestational diabetes based on clinical glucose tolerance tests and physician diagnosis.^2^ We used US standard reference data to calculate infant birth weight for gestational age (BWGA) *z* score.

Using logistic regression, we examined associations of wood stove use in each trimester with odds of (1) abnormal glycemia (impaired glucose tolerance or gestational diabetes) and (2) small for gestational age (SGA) (BWGA *z* score less than or equal to the 10th percentile). In secondary analyses, we used linear regression to examine associations of wood stove use with BWGA *z* score. We adjusted models for covariates listed in the Figure and present 2-sided *P* values. Data were analyzed from December 2018 to December 2019.

## Results

Of the 1223 women (median [IQR] age, 31.1 [28.2-34.4] years) included in this analysis, 536 (43.8%) reported using a wood stove during pregnancy; 135 (11.3%) had abnormal glycemia; and 125 (10.3%) had infants with SGA *z* scores. Women who used a wood stove (vs those who did not) were more likely to have a lower prepregnancy body mass index (calculated as weight in kilograms divided by height in meters squared; median [IQR], 24.0 [21.7-27.4] vs 24.8 [22.1-29.7]); greater neighborhood wood stove use (median [IQR], 20.8% (12.1%-28.9% vs 19.0% [9.9%-27.9%]); and live farther from a major roadway (median [IQR], 0.9 [0.2-2.1] km vs 0.8 [0.2-1.7] km) (Table).

**Table.** Characteristics of Participants in the New Hampshire Birth Cohort Study, Overall and by Home Wood Stove Use in Pregnancy^a^

Characteristic	Overall (N = 1223)	Wood stove use in pregnancy		*P* value^c^
		Yes (n = 536)^b^	No (n = 687)	
Maternal characteristics				
Age, median (IQR), y	31.1 (28.2-34.4)	31.3 (28.4-34.6)	31.0 (27.8-34.2)	.06
College graduate	829 (67.8)	370 (69.0)	459 (66.8)	.40
White	1189 (97.2)	522 (97.4)	667 (97.1)	.90
Married	1032 (84.4)	465 (86.8)	567 (82.5)	.05
Nulliparous	543 (44.4)	240 (44.8)	303 (44.2)	.80
Prepregnancy body mass index, median (IQR)^d^	24.4 (22.0-28.7)	24.0 (21.7-27.4)	24.8 (22.1-29.7)	<.001
Cohort enrollment season				
Spring	335 (27.4)	160 (29.9)	175 (25.5)	.10
Summer	300 (24.5)	135 (25.2)	165 (24.0)	.70
Fall	261 (21.3)	103 (19.2)	158 (23.0)	.13
Winter	327 (26.7)	138 (25.7)	189 (27.5)	.50
Abnormal glucose tolerance^e^	135 (11.0)	58 (10.8)	77 (11.2)	.95
Neighborhood characteristics, median (IQR)				
Wood stove use in census block, %^f^	19.6 (10.9-28.8)	20.8 (12.1-28.9)	19.0 (9.9-27.9)	.01
Home distance to nearest major roadway, km^g^	0.8 (0.2-1.9)	0.9 (0.2-2.1)	0.8 (0.2-1.7)	.04
Child characteristics				
Female sex	604 (49.4)	277 (51.7)	327 (47.8)	.20
Birth weight for gestational age *z* score, median (IQR)	0.14 (–0.48 to 0.72)	0.09 (–0.54 to 0.72)	0.16 (–0.43 to 0.72)	.20
Small birth weight for gestational age^h^	125 (10.2)	60 (11.2)	65 (9.5)	.40

Abbreviation: IQR, interquartile range.

^a^ Values are presented as number (percentage) unless otherwise specified.

^b^ A total of 32.5% of participants reported using a wood stove during the first trimester (396 of 1220 with first-trimester wood stove data), 34.6% during the second trimester (352 of 1017 with second-trimester wood stove data), and 31.4% during the third trimester (156 of 497 with third-trimester wood stove data).

^c^ *P* values are 2-tailed and compare characteristics between participants who used a wood stove during pregnancy vs those who did not. We used a χ^2^ test for all categorical variables and a *t* test for all continuous variables (except maternal age which we evaluated with a Wilcoxon rank-sum test because it was not normally distributed).

^d^ Calculated as weight in kilograms divided by height in meters squared.

^e^ Impaired glucose tolerance or gestational diabetes.

^f^ Per the 2011-2015 American Community Survey.

^g^ Primary or secondary road (ie, Feature Classification Code S1100 or S1200) in 2015 topologically integrated geographic encoding and referencing/Line database.

^h^ Less than the 10th percentile of birth weight for gestational age *z* score.

In adjusted models, women who used wood stoves in the first trimester (vs those who did not) had 1.52 times the odds (95% CI, 1.02-2.27) of abnormal glycemia during pregnancy. Women who used wood stoves in the third trimester (vs those who did not) had 1.81 times the odds (95% CI, 0.96-3.38) of having an SGA infant, and their infants had a 0.17 unit lower (95% CI, –0.36 to 0.01) BWGA *z* score. Wood stove use during other trimesters was not associated with glycemia or birth size (Figure).

**Figure.**
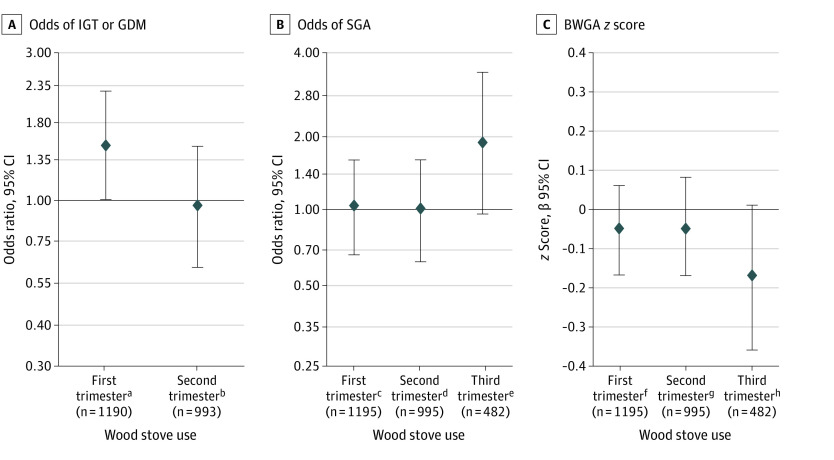
Covariate-Adjusted Associations of Household Wood Stove Use During Pregnancy Among Participants in the New Hampshire Birth Cohort Study A, Adjusted for maternal age (continuous), education (college graduate or not), race/ethnicity (white or other), marital status (married or not), parity (nulliparous or not), prepregnancy body mass index (continuous), cohort enrollment date (continuous), cohort enrollment season (continuous sine and cosine functions of enrollment date), and neighborhood wood stove use (continuous). We considered but did not include the following covariates that did not confound the exposure-outcome association (ie, change effect estimate by >10%): smoking in current or preceding trimester, alcohol use in current or preceding trimester, and home distance to nearest major roadway. B and C, Adjusted for maternal age (continuous), education (college graduate or not), race/ethnicity (white or other), prepregnancy body mass index (continuous), cohort enrollment season (continuous sine and cosine functions of enrollment date), neighborhood wood stove use (continuous), home distance to nearest major roadway (continuous), and child sex (dichotomous). We considered but did not include the following covariates that did not confound the exposure-outcome association (ie, change effect estimate by greater than 10%): maternal marital status, parity, smoking in current or preceding trimester, alcohol use in current or preceding trimester, and cohort enrollment date. GDM indicates gestational diabetes; IGT, impaired glucose tolerance; SGA, small for gestational age. ^a^OR (odds ratio), 1.52 (95% CI, 1.02-2.27); *P* = .04. ^b^OR, 0.98 (95% CI, 0.62-1.52); *P* = .92. ^c^OR, 1.03 (95% CI, 0.67-1.55); *P* = .89. ^d^OR, 1.00 (95% CI, 0.63-1.56); *P* = .99. ^e^OR, 1.81 (95% CI, 0.96-3.38); *P* = .06. ^f^β = –0.05 (95% CI, –0.17 to 0.06); *P* = .38. ^g^β = –0.05 (95% CI, –0.17 to 0.08); *P* = .45. ^h^β = –0.17 (95% CI, –0.36 to 0.01); *P* = .07.

## Discussion

To our knowledge, this is the first US study to evaluate the health outcomes associated with wood smoke exposure during pregnancy, and our findings align with the growing literature showing that traffic-related air pollution may be associated with maternal glycemia and birth size.^3,4^ Wood stoves and traffic emit similar pollutants (eg, black carbon, which was over 70% higher in homes with a wood stove in a prior analysis of this cohort).^5^ Air pollutants increase inflammation, which may exacerbate insulin resistance in early pregnancy and cause placental inflammation and impaired maternal-fetal nutrient transport in later pregnancy.^6^ Generalizability is a limitation of our analysis, as our cohort is largely white and from a rural region. Also, our sample size is limited, particularly for analyses of third-trimester wood stove use, which we did not record until 2014. Wood stove use exposes pregnant women to high levels of indoor air pollution, and use of wood stoves for heat is increasing in the United States. Thus, our findings highlight the importance of continued study of domestic wood stove use during pregnancy on mother and infant health.
